# Supplementary figures and images for: The risks of using the chi-square periodogram to estimate the period of biological rhythms
Source: PLoS Comput Biol. 2021 Jan 6;17(1):e1008567. doi: 10.1371/journal.pcbi.1008567 (PMC7815206; doi:10.1371/journal.pcbi.1008567)

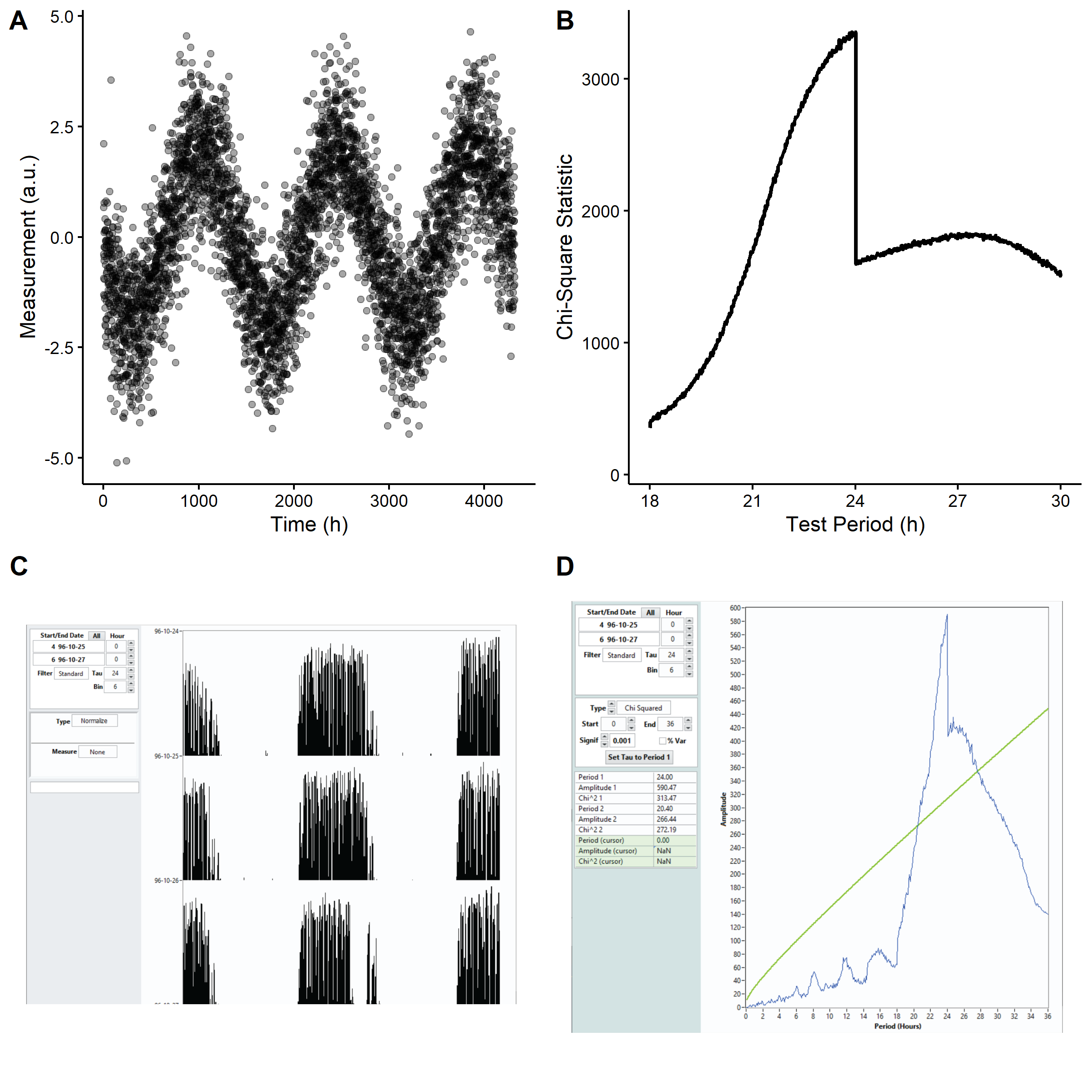

Supplement: S1 Fig — (A) A simulated time-course with a sinusoidal rhythm of amplitude 2. (B) The corresponding chi-square periodogram calculated by the xsp R package. (C) ClockLab actogram view of the pre-loaded “Sample 1” dataset from days 4 to 6 and (D) the corresponding chi-square periodogram. (TIF) [file pcbi.1008567.s001.tif]

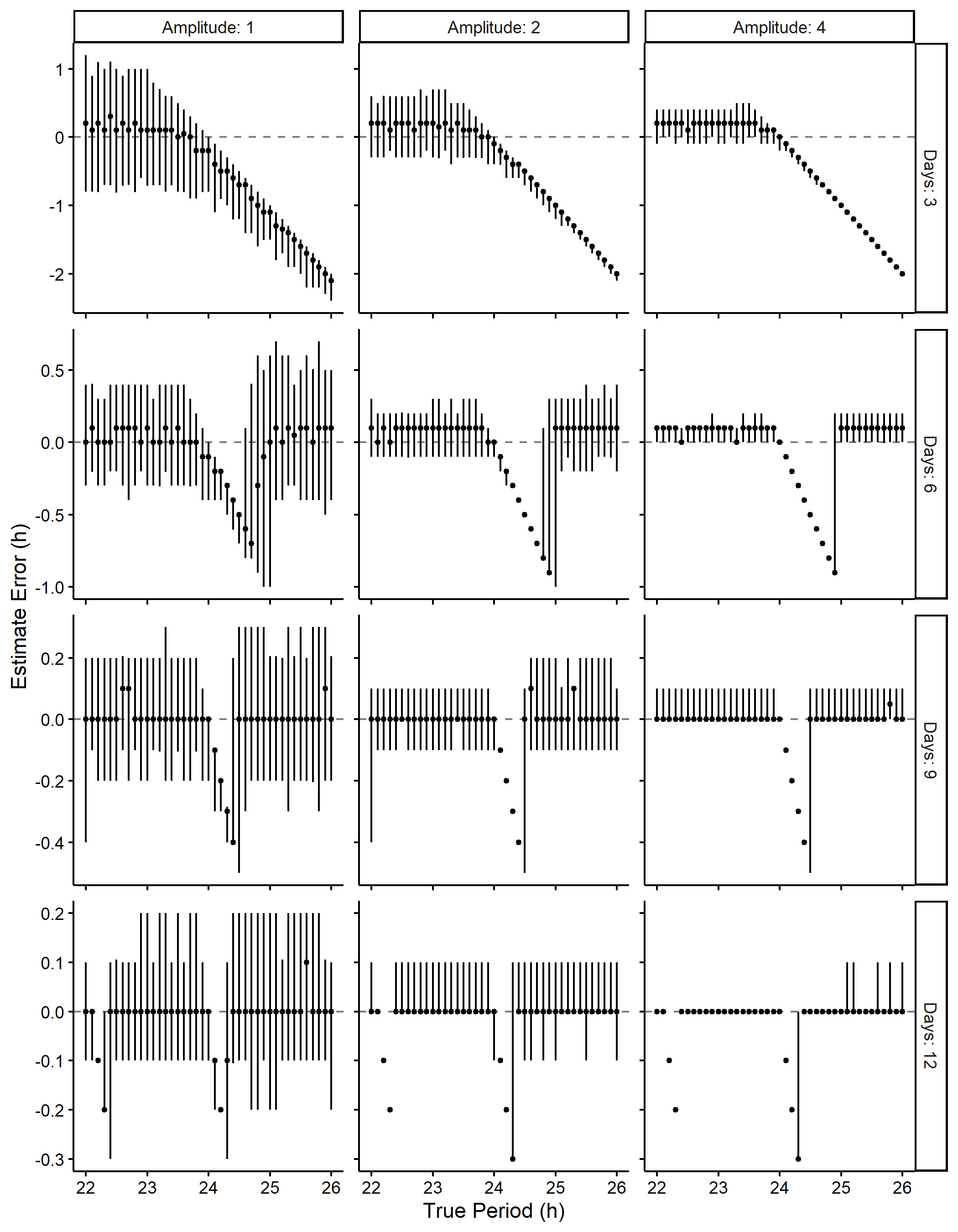

Supplement: S2 Fig — Estimate error on simulated time-courses of various lengths having a sinusoidal rhythm with various values of amplitude and true period. Each point represents the median of 100 time-courses, and each vertical line represents the 5th-95th percentile range. (TIF) [file pcbi.1008567.s002.tif]

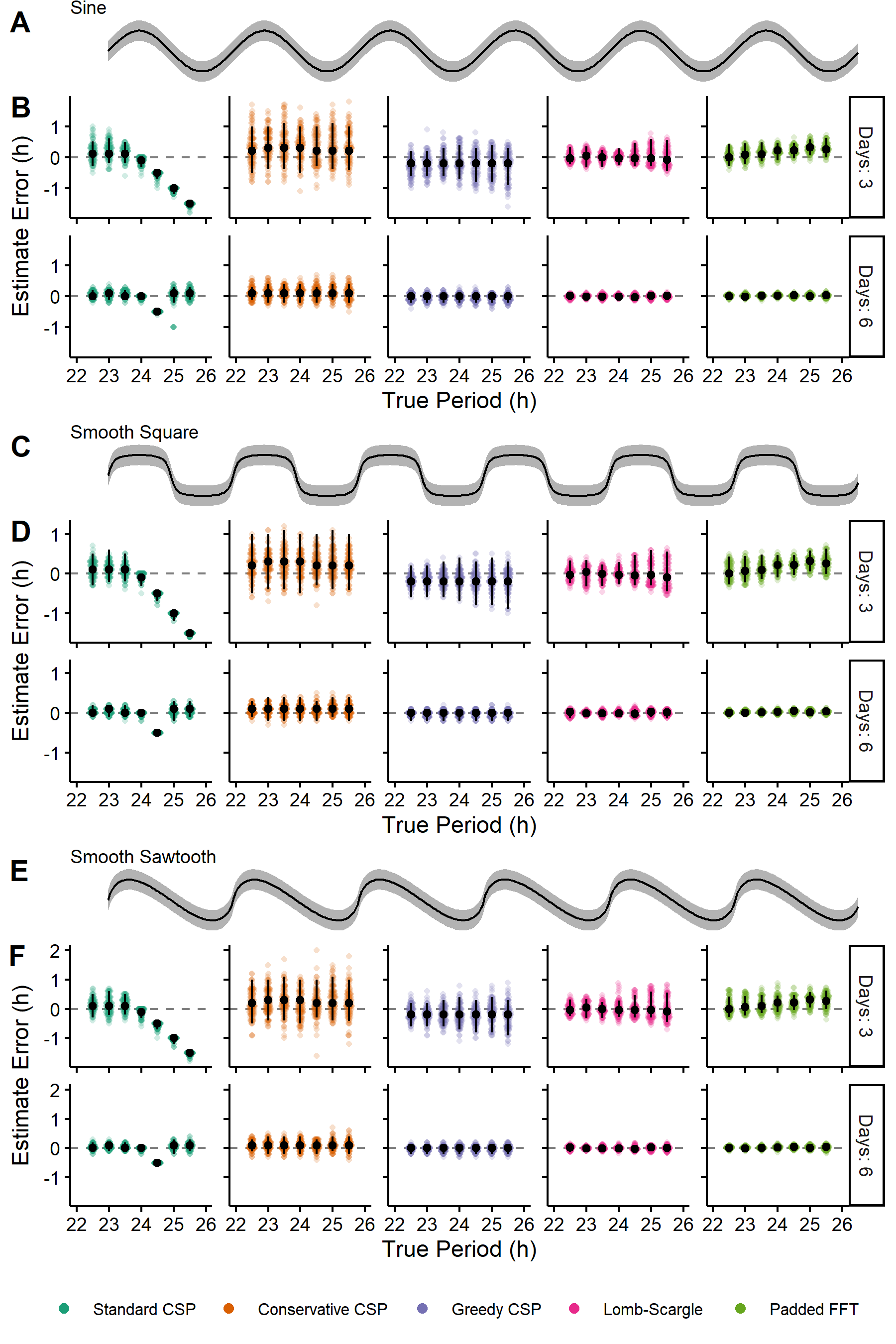

Supplement: S3 Fig — Waveforms of (A) sinusoidal, (C) smooth square, and (E) smooth sawtooth rhythms of amplitude 2. Black curves indicate expected rhythm, grey regions indicate one standard deviation of the Gaussian noise above and below. Estimate error for each method on simulated time-courses of various lengths and having a (B) sinusoidal, (D) smooth square, or (F) smooth sawtooth rhythm. Each point represents a simulated time-course, with 100 time-courses per combination of length and true period. Black circles and vertical black lines represent the median and 5th-95th percentile range, respectively. (TIF) [file pcbi.1008567.s003.tif]

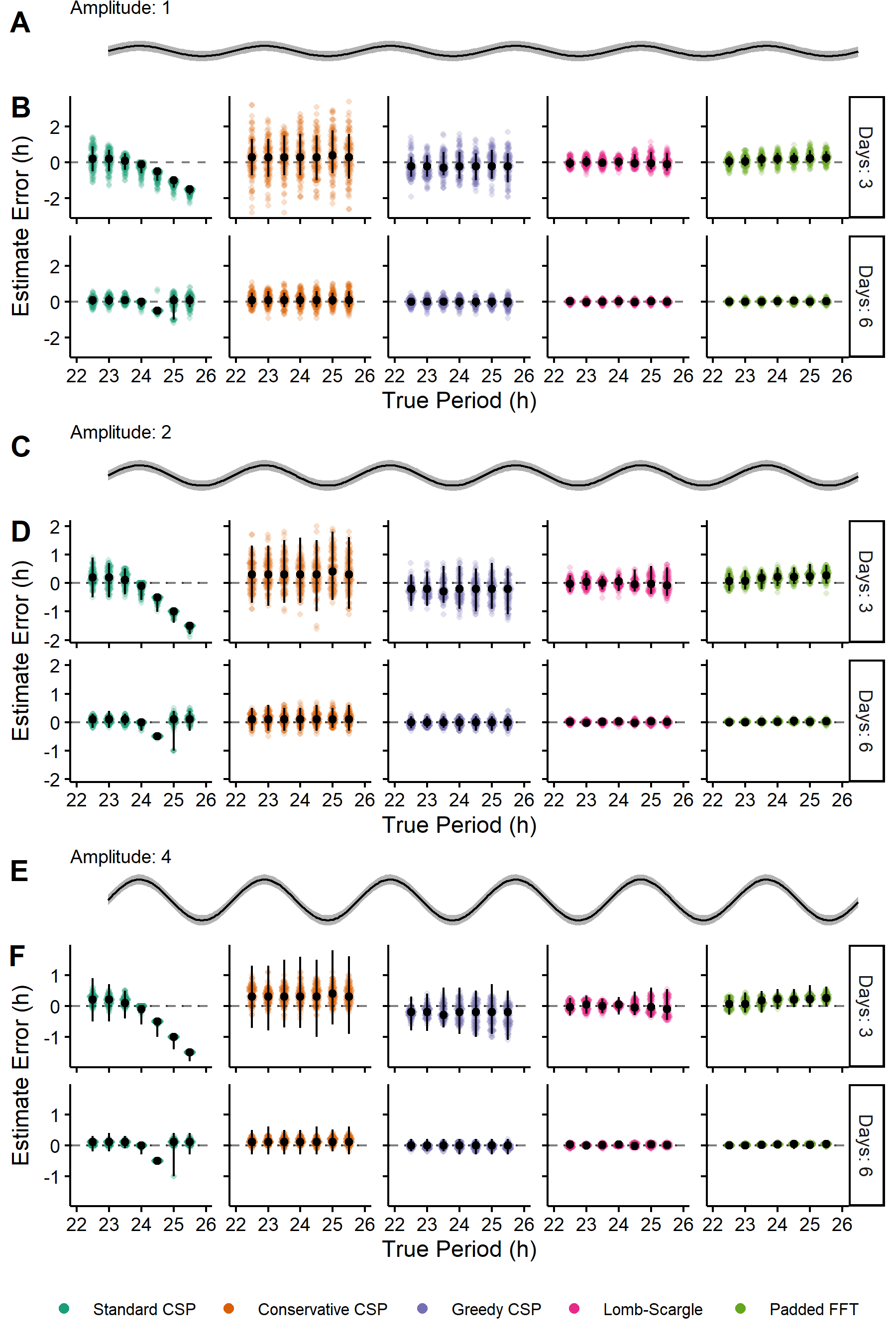

Supplement: S4 Fig — Sinusoidal rhythms of amplitude (A) 1, (C) 2, and (E) and 4. Black curves indicate expected rhythm, grey regions indicate one standard deviation of the Gaussian noise above and below. Estimate error for each method on simulated time-courses of various lengths and having a rhythm with amplitude (B) 1, (D) 2, or (E) 4. Each point represents a simulated time-course, with 100 time-courses per combination of length and true period. Black circles and vertical black lines represent the median and 5th-95th percentile range, respectively. (TIF) [file pcbi.1008567.s004.tif]

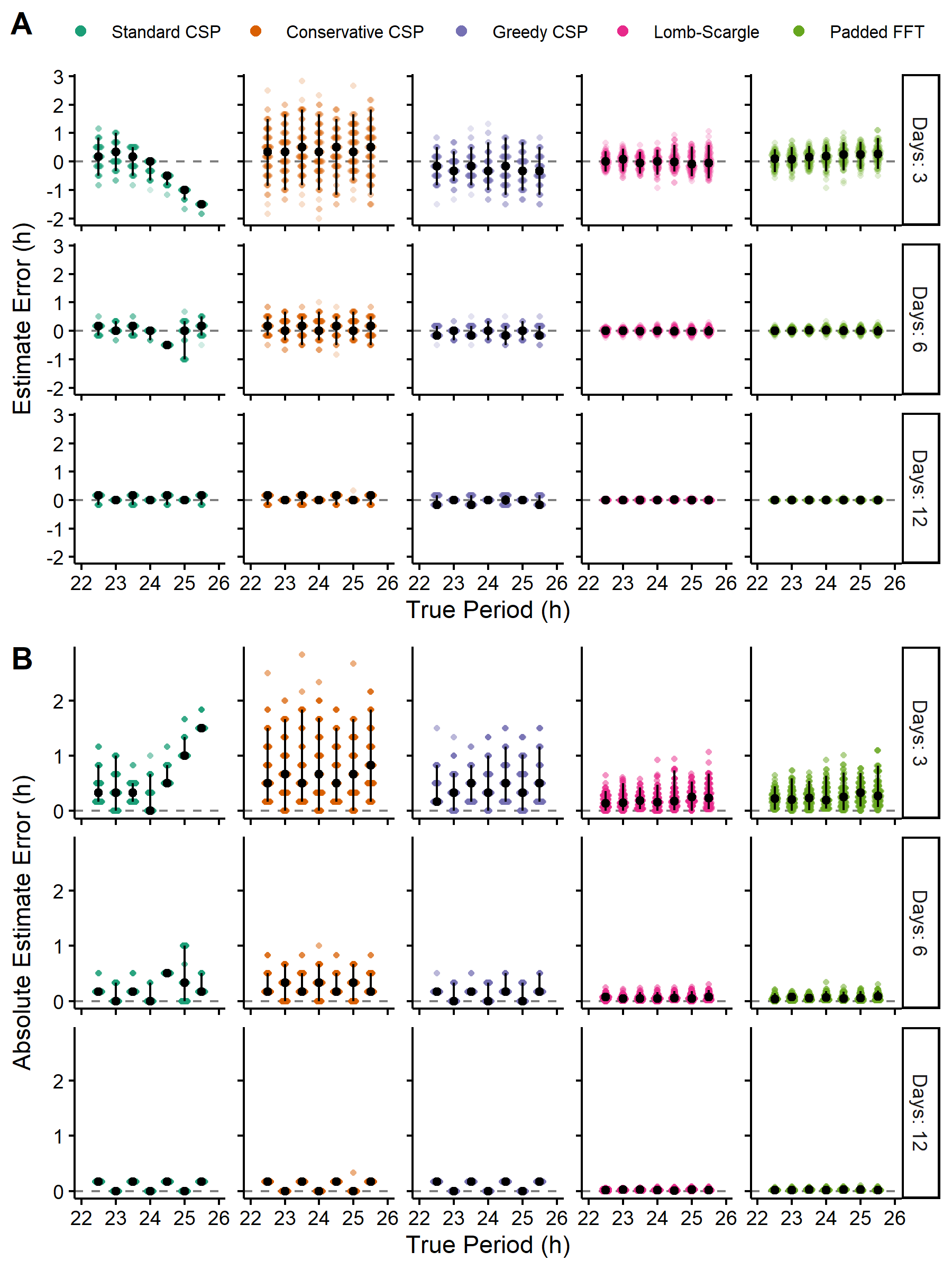

Supplement: S5 Fig — (A) Estimate error and (B) absolute estimate error for various methods on simulated time-courses of various lengths and with various values of true period. Each point represents a simulated time-course, with 100 time-courses per combination of length and true period. Each time-course had a sinusoidal rhythm with amplitude 2. Black circles and vertical black lines represent the median and 5th-95th percentile range, respectively. (TIF) [file pcbi.1008567.s005.tif]

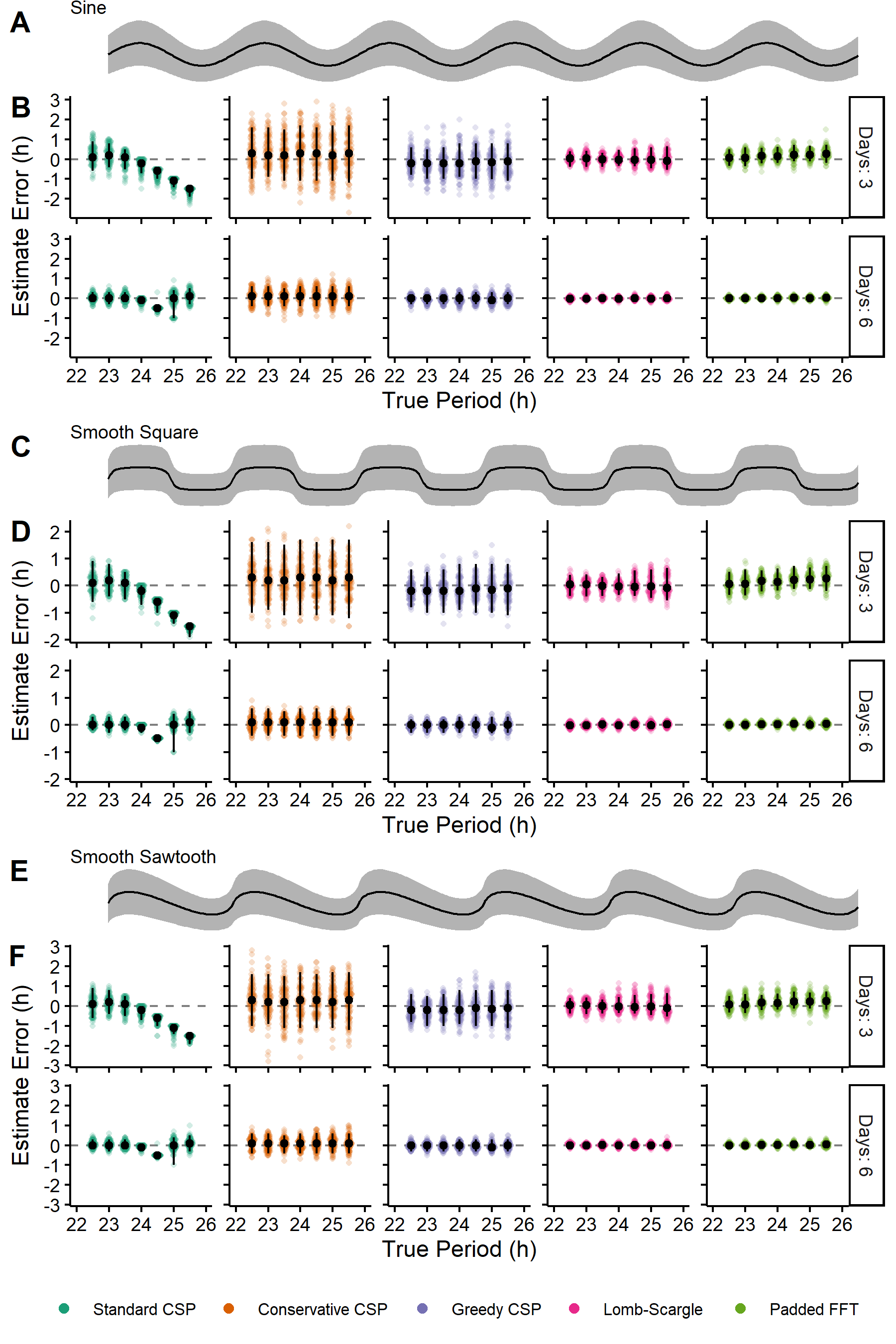

Supplement: S6 Fig — Waveforms of (A) sinusoidal, (C) smooth square, and (E) smooth sawtooth rhythms of amplitude 2. Black curves indicate expected rhythm, grey regions indicate one standard deviation above and below. Estimate error for each method on simulated time-courses of various lengths and having a (B) sinusoidal, (D) smooth square, or (F) smooth sawtooth rhythm. Each point represents a simulated time-course, with 100 time-courses per combination of length and true period. Black circles and vertical black lines represent the median and 5th-95th percentile range, respectively. (TIF) [file pcbi.1008567.s006.tif]

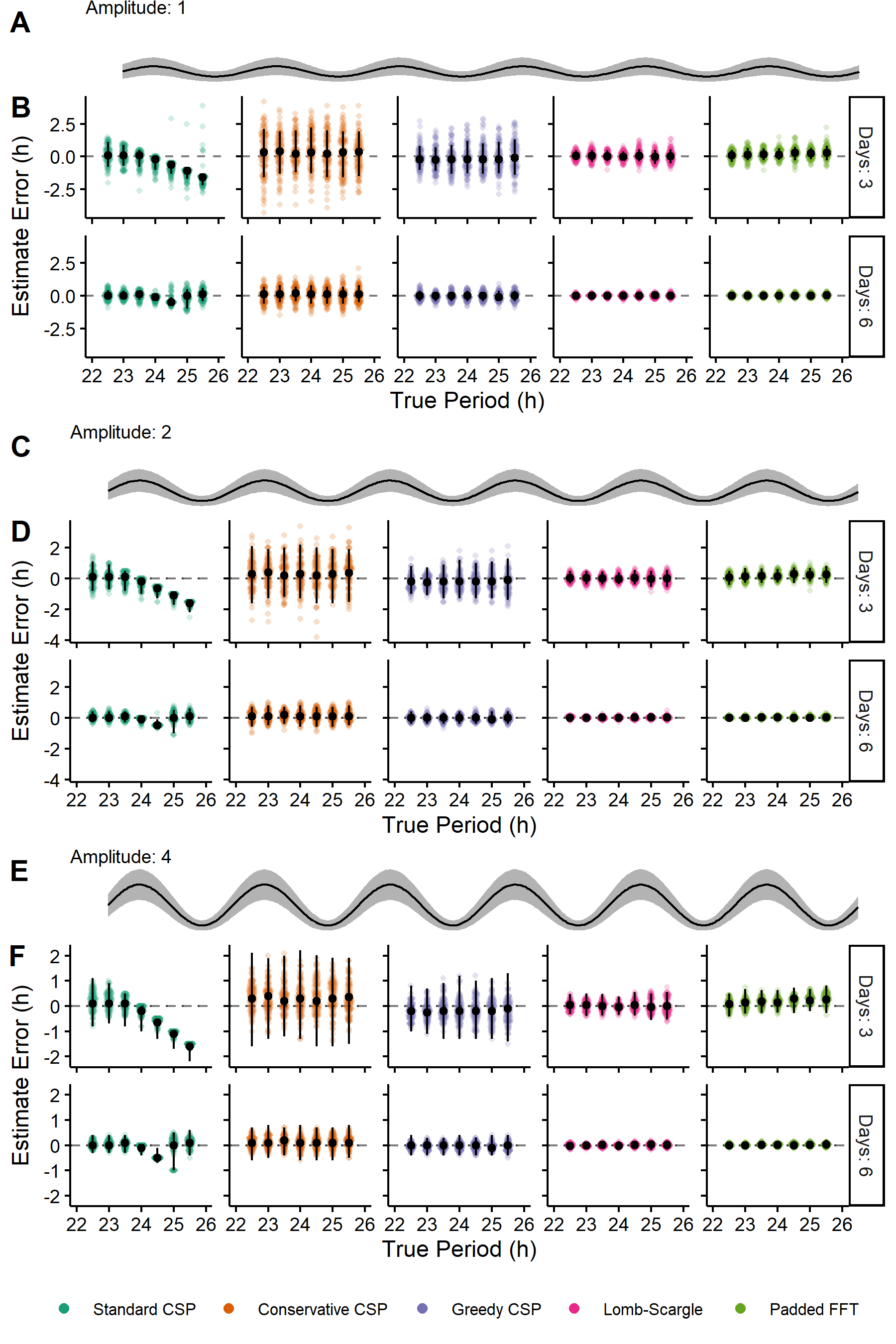

Supplement: S7 Fig — Sinusoidal rhythms of amplitude (A) 1, (C) 2, and (E) and 4. Black curves indicate expected rhythm, grey regions indicate one standard deviation above and below. Estimate error for each method on simulated time-courses of various lengths and having a rhythm with amplitude (B) 1, (D) 2, or (E) 4. Each point represents a simulated time-course, with 100 time-courses per combination of length and true period. Black circles and vertical black lines represent the median and 5th-95th percentile range, respectively. (TIF) [file pcbi.1008567.s007.tif]

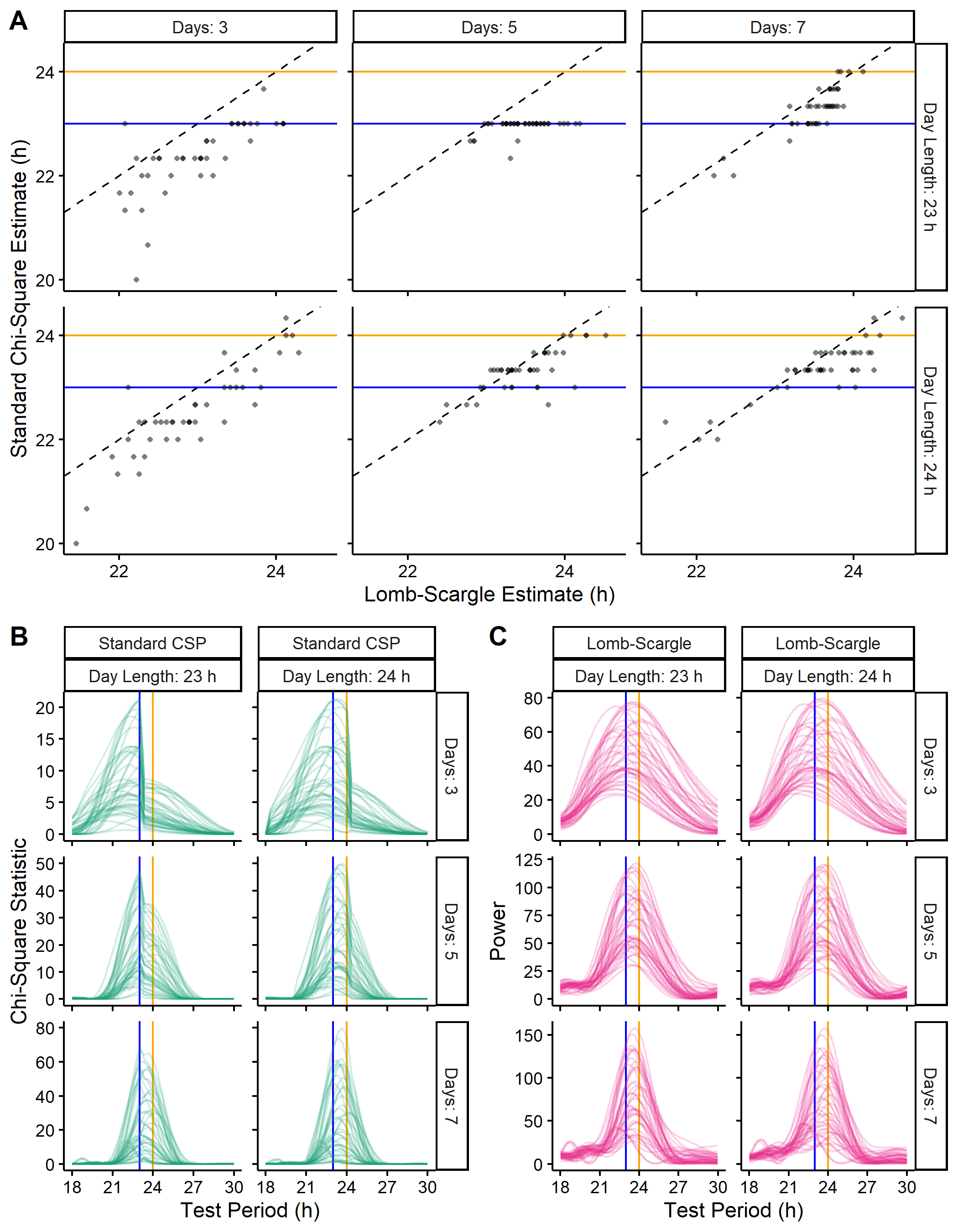

Supplement: S8 Fig — (A) Scatterplots of estimated period for the LSP and standard CSP on time-courses truncated to various lengths based on various numbers of days and day lengths. Periodograms for the (B) standard CSP and (C) LSP on the same truncated time-courses. Blue and orange lines indicate 23 and 24 h, respectively. (TIF) [file pcbi.1008567.s008.tif]
