# Supplementary material for: The risks of using the chi-square periodogram to estimate the period of biological rhythms
Source: PLoS Comput Biol. 2021 Jan 6;17(1):e1008567. doi: 10.1371/journal.pcbi.1008567 (PMC7815206; doi:10.1371/journal.pcbi.1008567)
Supplement: Attachment — Submitted filename: period estimate response to reviewers - review commons 1.pdf [file pcbi.1008567.s015.pdf]

We thank the reviewers for their feedback and encouragement. We have now fully revised the manuscript to address all comments. Our specific responses are provided below and we have highlighted changes in the text. The major additions are:

- analysis of simulated time-courses with lower temporal resolution
- analysis of ex vivo PER2::LUCIFERASE SCN recordings
- analysis of simulated time-courses with Poisson distributions of noise
- plotted summary statistics for several figures
- mathematical formula and explanation in the Methods

Overall, these revisions have strengthened our findings and improved the manuscript, particularly in demonstrating that the issues with the chi-square periodogram are not specific to sampling interval or data type.

Reviewer #1 (Evidence, reproducibility and clarity (Required)):

Summary:

Tackenberg & Hughey investigate the reliability of a popular period estimation algorithm, the chi-square periodogram. They find a bias in the estimation, and through careful investigation identify the cause. This is a well executed and well presented study.

Comments:

In Figs 2+3 the authors show that the discontinuity in periodogram coincides with the number of complete cycles, K. However, in Fig 2C there are several other positions where K abruptly changes, but little effect on the chi-squared statistic is observed. Can the authors offer an explanation as to why the magnitude of the discontinuities differ?

We have taken a closer look at how each component of the chi-square statistic calculation changes at points where K decreases, and have found that discontinuities do always occur at these points. In addition to the obvious effect of the $K * N$ term on the sudden decreases, we found that the sum of squares of the column means alone (the primary component of the numerator) also changes abruptly at each transition point of K. As a result, the discontinuity magnitude is likely roughly proportional to the amplitude of the chi-square statistic at that point.

An important claim is that the discontinuity is observed in multiple software implementations. However, the plots of Supplementary Fig 1C,D are presented too small to evaluate this claim.

In Supplemental Fig. 1C-D, the critical information is the shape of the periodogram and the presence of a discontinuity, so we believe the plot sizes are appropriate.

It may be of interest to apply the algorithms to a single-cell experimental data set which are qualitatively different (e.g., oscillation shape, damping).

We have created a new supplemental figure (Supplemental Fig. 8) by applying the strategy and visualization used in Fig. 6 to SCN PER2::LUC recordings instead of wheel-running data, and have updated the text accordingly.

Reviewer #1 (Significance (Required)):

It has been previously shown that the chi-square periodogram algorithm has performance shortcomings for the analysis of circadian data (e.g. Zielinski et al., 2004). However, this study demonstrates exactly why, giving more conclusive evidence to support the conclusion that it should be avoided. This will be useful to many in the mammalian circadian community. It should be noted however that other algorithms are already favoured by other ciock communities (e.g. plant), even if a rigorous understanding of the biases were lacking.

The methods developed here will be valuable for future comparisons of circadian algorithms. Of particular importance will be comparing algorithms for analysis of single-cell rhythms or non-stationary rhythms.

Reviewer #2 (Evidence, reproducibility and clarity (Required)):

Chi-squared periodograms (CSP) are routinely used in circadian biology. In particular, this test has been used to determine circadian period in behavioral data (e.g. actigraphy) in mammals, flies and other species. This paper suggests that CSP, in some circumstances (e.g. where there are discontinuities), that CSP could be improved by changing the algorithm. They propose different steps to do this (e.g. using their greedy CSP code) and/or by using alternative tests such as Lomb-Scargle.

The authors use simulated data to demonstrate their findings, and whilst I can see the benefits of this, it would be useful to benchmark the algorithms on actual real world circadian data (e.g. actograms from mouse or fly experiments). Although these types of data may not be publicly available, it would be highly likely to be available from multiple labs in the circadian field. In particular, fly datasets will be abundant in many clock labs. This would aid the utility of the papers findings for the field.

Fig. 6 is entirely based on real-world circadian data (mouse wheel-running activity), as is the newly added Supplemental Fig. 8.

Reviewer #2 (Significance (Required)):

The paper is helpful for the circadian field when dealing with datasets that may contain discontinuities.

It appears that the paper will be primarily useful for behavioral data, rather than, for example, transcriptomic time courses, since these tend to be much shorter and less sample intensive. Thus, it would be useful for circadian (and other) researchers analysing activity data in particular.

My expertise is in circadian rhythms, both behavioural and molecular (e.g. sequencing) level analyses. Thus, I would be a possible end-user for the algorithms in this paper.

Reviewer #3 (Evidence, reproducibility and clarity (Required)):

Summary:

The authors identify a serious flaw in a popular method called Chi-squared periodogram (CSP) for period estimation in circadian rhythms. They systematically get to the source of the problem -- a discontinuity in the test statistic. This flaw leads to a bias in the period estimate. They present two modifications to the CSP, one of which they prefer. Nevertheless, they show that

other more flexible methods such as Lomb-Scargle Periodogram work well without this discontinuity (bias) issue.

Major Comments:

1.One thing the authors do not include is timeseries lengths of non-integer days. Would it not be an interesting suggestion to choose a non-integer length time course, which is not a multiple of the periods of interest, and still continue using CSP as is ? This is also rather counter-intuitive.

Figs. 3A and 6 and newly added Supplemental Fig. 8 use non-integer (24-h) days.

2.I suppose the authors use a sampling resolution of 6min with wheel-running activity in mind. But it would be worth it in the interest of completeness to also consider a lower resolution. There is nothing in this study that ties it to the specific application, is it not?

Although a sampling resolution of 6 minutes is not specific to wheel-running activity, we have added an analysis identical to that of Fig. 5 but with a resolution of 20 minutes (Supplemental Fig. 5). Additionally, the PER2::LUC SCN recordings analyzed in Supplemental Fig. 8 have a sampling resolution of 20 minutes.

3.The authors discuss only the mean absolute error in the text but isn't the direction (sign) of the error also of interest. As far as I can see in Fig 5, conservative CSP overestimates and greedy CSP generally underestimates periods.

We discuss both the error (references to Fig. 5A) and absolute error (references to Fig. 5B) in the text. We feel the interpretation suggested by the reviewer may be too reliant on the results of 3-day simulations, as the apparent underestimation by greedy appears far less substantial in simulations of 6 and 12 days.

Minor Comments:

1.I would like to see the formulae for the ratio of variances and p-values to be clear about how the authors computed the CSP. They describe it in words already, but I think some mathematics is warranted here.

We have added the formula for the standard chi-square periodogram to the Methods section.

2.It is nice to the see the raw data in the plots. But I would like to see the plot of the summary statistics (mean and variance/st. dev) for each of scatter plots to judge the size of bias. It is not easy to do this with the Excel sheet.

We have overlaid a black circle representing the median and a vertical black line representing the 5th-95th percentile range onto Fig. 5 and Supplemental Figs. 3-7.

Reviewer #3 (Significance (Required)):

The authors present a sobering perspective on the chi-squared periodogram, which is still very popular among empirical biologists. They plainly show using artificial data that it is better to avoid the CSP when possible, although they suggest improvements to the CSP. The authors provide an R package to perform the analysis.

There have been previous work that have highlighted other limitations of the CSP. This might be considered one more nail in the coffin of the CSP.

I think this paper would be interest to both computational biologists and wet-lab biologists, but I think it ought to have a greater influence on the latter as the former already resort to more sophisticated approaches.

My expertise is in Computational and Theoretical biology.
